# Present-day breeding of legumes and groat crops in Russia

**DOI:** 10.18699/VJ21.041

**Published:** 2021-07

**Authors:** V.I. Zotikov, S.D. Vilyunov

**Affiliations:** Federal Scientific Center of Legumes and Groat Crops, Orel, Russia; Federal Scientific Center of Legumes and Groat Crops, Orel, Russia

**Keywords:** legumes and groat crops, breeding, pea, soy, buckwheat, millet, variety, зернобобовые и крупяные культуры, селекция, горох, соя, гречиха, просо, сорт

## Abstract

The production of pedigree seeds is not only an important but also a cost-effective means of increasing the yield and efficiency of agriculture. The genetic potential of varieties can be unlocked only by choosing those adaptive to the soil and climatic conditions in a particular region, using modern tools for plant protection, and applying balanced mineral nutrition. These are the most important factors determining the performance. In the course of breeding and genetic work, the Federal Scientific Center of Legumes and Groat Crops (FSC LGC) has created new soybean varieties, whose high biological and economic potentials are combined with resistance to stress factors. Despite the close relationship between productivity and growing season duration, the highly productive and early-ripening (100–115 days) soybean varieties raised at FSC LGC can yield 2.5 to 3.5 t/ha, the grain having high contents of protein (37–42 %) and fat (18–22 %), depending on the climatic conditions in a particular year of cultivation. They are less temperature-sensitive than other domestic or foreign varieties. It is important that our soybean varieties are not genetically modified. New pea varieties created at FSC LGC in 2015–2020 differ in growing season duration and morphological features. They are adaptable to the soil and climatic conditions of a region, which ensures the maximum realization of their potential. The main factor in increasing yields and stabilizing the production of buckwheat and millet grain in the Russian Federation is the creation and adaption of new earlyripening and high-yielding varieties of the determinate type adapted to the specific natural and climatic conditions of different regions of Russia.

## Abstract (ru)

Производство сортовых семян – не только важный, но и экономически выгодный ресурс повышения урожайности и эффективности сельскохозяйственной отрасли. Реализация генетического потенциала сортов возможна лишь при условии внедрения в производство сортов, адаптивных к почвенно-климатическим условиям региона, использовании при их возделывании современных средств защиты растений, сбалансированного минерального питания, являющихся важнейшими факторами, определяющими уровень урожайности. В процессе селекционно-генетических работ в Федеральном научном центре зернобобовых и крупяных культур (ФНЦ ЗБК) созданы новые сорта сои с высокой биологической и хозяйственной продуктивностью, устойчивые к основным стрессовым факторам. Несмотря на тесную взаимосвязь между продуктивностью и длиной вегетационного периода, полученные в ФНЦ ЗБК скороспелые (100–115 сут) высокопродуктивные сорта сои способны давать урожай порядка 2.5–3.5 т/га с высоким содержанием белка (37–42 %) и жира (18–22 %) в зависимости от условий конкретного года возделывания. Они менее требовательны к теплу, чем другие сорта российской и зарубежной селекции. Важно, что эти сорта сои не являются генетически модифицированными. Новые сорта гороха, выведенные в ФНЦ ЗБК в период с 2015 по 2020 г., отличаются по длине вегетационного периода, морфологическим признакам, обладают адаптивностью к почвенно-климатическим условиям региона, что обеспечивает максимальную реализацию потенциала их урожайности. Основной фактор увеличения урожайности и стабилизации производства зерна гречихи и проса в Российской Федерации – создание и ускоренное использование в производстве новых скороспелых и высокоурожайных сортов детерминантного типа, адаптированных к конкретным природно-климатическим условиям различных регионов страны

## Introduction

Legumes and groat crops play an important role in providing people with high quality food and animal husbandry with fodder. Their main advantage is a high content of proteins and essential amino acids. In addition, leguminous crops play an important environment-forming role in crop rotations, providing the soil with 30 to 90 kg of nitrogen per hectare. At high fertilizer prices, they can significantly increase the profitability of grain crops grown after legume predecessors. 

The areas under soybean (Glycine max (L.) Merrill) in the Russian Federation increase every year, and a significant leap occurred in 2017–2018. In general, over the past five years, they increased by 50 %. The structural changes in the regions of soybean cultivation involve, first of all, the Far Eastern Federal District of Russia (Sinegovskaya, 2021). Due to climatic conditions, the Far East occupies the main share in the structure of soybean planting areas. The Belgorod, Kursk, and Orel oblasts are in the lead in soybean cultivation in the Central Federal District. They comprise 58 % of the soybean hectarage in the district.

The land sown with the pea (Pisum sativum L.) in the Russian Federation fluctuates from year to year within 1.5–1.7 million hectares, and all legumes (other than the soybean) occupy 2.0–2.5 million hectares. Over the past 10 years, soybean crops in Russia have increased fivefold on the average and doubled the area of pea crops, and in terms of gross grain yield they doubled the total amount of all other leguminous crops grown in Russia. In the structure of leguminous crop (other than soybean) production, 74 % is constituted by the pea, 12 % by the chickpea (Cicer arietinum L.), 7 % by lupine (Lupinus), and 6 % by the common vetch (Vicia sativa L.). By geography of the Russian Federation, the Stavropol and Altai Territories and the Rostov Region are in the lead in pea hectarage.

In the Russian Federation, the pea and soybean have the greatest production value among leguminous crops, whereas buckwheat (Fagopyrum esculentum Moench) and millet (Panicum miliaceum L.) dominate among groat crops. Russia ranks second in the world in the hectarage and gross grain yield of peas 

About 40 research institutions located in different regions of Russia are engaged in the breeding of legumes and groat crops, according to the “Interdepartmental coordination plan for fundamental and applied research on the scientific support of the agro-industrial complex of the Russian Federation for 2016–2020”. The Federal Scientific Center of Legumes and Groat Crops (FSC LGC) is the coordinator of this work. In the course of introduction of modern genetic and biotechnological breeding methods in this period, over 80 varieties of legumes and groat crops were conveyed to the State variety testing, including 32 varieties of the common pea, 5 varieties of vetch and lentil, 6 varieties of chickpea, 6 varieties of common bean, 8 varieties of buckwheat, and 8 varieties of millet. Of them, 60 varieties were patented.

## Pea breeding

Scientists at FSC LGC employ an effective technique, which accelerates pea breeding to improve symbiotic nitrogen fixation. In this technique, a host plant is obtained by hybridization of initial forms, one of which bears the recessive sym2 gene. This gene determines the resistance of peas to local and master seed strains of nodule bacteria (Rhizobium leguminosarum bv. viciae). The progeny of the F1 and F2 hybrids is propagated on a nitrogen-free background and inoculated with a master seed strain of nodule bacteria. Plants that do not enter the symbiosis with nodule bacteria and, as a consequence, remain unable to fix atmospheric nitrogen, are colored yellow. All green plants are removed from the plot. Then mineral nitrogen is added to the substrate, on which etiolated hybrid pea plants are grown in a normal way, and genotypes homozygous for the sym2 gene develop. After that, the first backcross is carried out. After a series of backcrosses and selection on the nitrogenfree background, the selected genotypes are propagated and tested for the effectiveness of interaction with a certain strain of nodule bacteria.

**Fig. 1.**
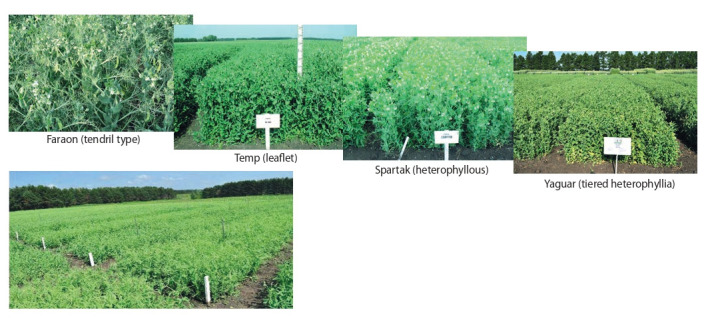
New morphotypes of peas used in industrial production in the Russian Federation.

Pea breeding is aimed at increasing the productivity and quality of grain by improving the morphotype of plants, primarily by restructuring the architectonics of the leaf apparatus (Fig. 1). Modern varieties have changed not only the leaf apparatus but also the plant habit. The main result of this direction is the creation of leafless, or tendril varieties (see Fig. 1), which ensure the resistance of the agrocenosis to lodging. In this way, the complex task of increasing the technological effectiveness of pea cultivation and reducing grain loss during harvesting was solved.

Along with the solution of the tasks related to the adaptability and technological effectiveness, FSC LGC breeders created and put into production pea varieties combining the tendril leaf type and the determinate (self-limited) type of stem growth, which ensures uniform ripening of beans in different tiers to facilitate harvesting by direct combining. Currently, more than 75 % of such varieties are used in production.

A promising direction in pea breeding is the creation of heterophyllous varieties, or chameleons, characterized by layered variability of leaves and high resistance to lodging due to the transformation of leaf blades into tendrils, which hold plants in an upright position till their technical ripeness (Zadorin, 2013; Zelenov et al., 2018).

The first domestic variety obtained by FSC LGC breeders was the Spartak variety with longline heterophyllia (see Fig. 1), and in 2020, another variety of similar morphotype, Jaguar, was included in the “State Register of Selection Achievements Authorized for Use for Production Purposes” (see Fig. 1). Since 2019, two more leafless pea varieties, Estafeta and Biryuza, are under State testing. The latter is for marketing green. It surpasses all previous varieties bred at FSC LGC in protein content (26– 27 %). Breeders have obtained forms with protein contents within 30–32 %. This direction of pea breeding is promising for creating varieties intended for deep processing of peas into protein isolates to produce essential amino acids.

## Soybean breeding

In modern agriculture, the soybean is becoming a key legume crop in crop rotation, which is of great national economic importance. Soybeans contain 35–45 % protein of high quality in terms of amino acid composition, solubility, and digestibility; 17–25 % oil suitable for food, feed, and technical purposes; 20–30 % carbohydrate compounds, including 10–12 % soluble sugars; 5–6 % ash mineral macro- and microelements; 12 essential vitamins and a range of other nutrients.

In general, as evidenced by research data, domestic soybean varieties, including those created at FSC LGC, are not inferior to foreign analogues. 

Presently, FSC LGC possesses eight soybean varieties of different maturity groups with growing seasons within 105–115 days, i. e., all varieties in the Central Black Earth Region ripening in late August – early September. They allow the soybean, like pea, to be used in crop rotations as a good precursor for winter cereals. 

In 2018–2019, the breeding material of FSC LGC soybeans was significantly expanded due to collection plantings, including the most productive foreign varieties with high protein contents. The expansion of the working collection of soybean varieties made it possible to include them in the breeding process. Attention was focused on varieties with yields of 3–5 t/ha and protein contents of at least 40 %.

**Fig. 2.**
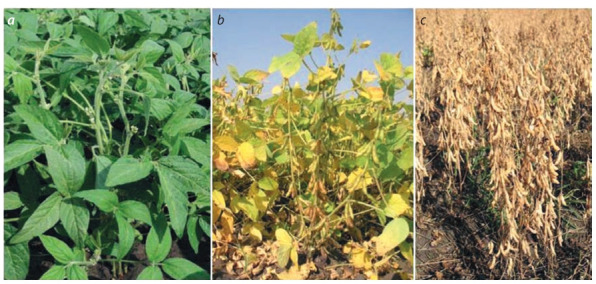
A soybean variety of the determinate type from FSC LGC in different phases of development: (a) budding; (b) ripening of beans; (c) harvesting period.

This year, 38 FSC LGC soybean lines are under comparative and approval tests. In 2020, a new soybean variety Shatilovskaya 17 was included in the “State Register of Selection Achievements Authorized for Use for Production Purposes” (2020). The variety is early ripening, determinate type (Fig. 2). Its yield in plots of Orel oblast averaged over three years was 2.65 t/ha (maximum 3.73 t/ha), which exceeded the standard value by 0.31 t/ha, and the protein content in the grain was 37–40 %.

The primary role in increasing legume yield and bean quality is played by breeding methods that involve search for sources and the creation of donors of economically important traits for new varieties with specified commercially important parameters. Therefore, long-term breeding programs have been developed for soybean growth in a number of regions of Russia to increase yields and improve the consumer and technological qualities of soybeans. By using a promising technology of soybean cultivation, genotypes of this crop that combine high adaptivity with breeding value were identified. In general, trends in soybean breeding will be aimed at increasing productivity to at least 3.5–4.0 t/ha and protein content in beans to 40–43 % by means of the creation of single-stem and branching varieties of the determinate type with a highly developed generative sphere.

The northern boundaries of the soybean range will be expanded with the creation of new varieties characterized by a weak or neutral photoperiodic response, soil and air temperature tolerance, and responsiveness to inoculation with various strains of rhizobia. Varieties are being created with high bean quality indicators: protein content, fat content, and suitability for deep processing to obtain protein isolates and oil. It is important to create varieties with high adaptability to environmental stress factors, high air temperature during the flowering period, and water shortage in the budding phase. In connection with the expansion of soybean planting areas, it is advisable to make a point of breeding for plant resistance to major pests and diseases, whose spectrum will undoubtedly grow in connection with global and local climate changes.

With regard to the significant climate changes, the associated increase in temperature and aridity, and more frequent extreme events, one should choose a proper approach to the selection of crops, their maturity types, and the development of varietal technologies that would allow reaching the productivity potential and quality inherent in new varieties. For example, the productivity potential of the pea and soybean varieties cultivated in Russia is not reached in full. The main causes are the slow introduction of new varieties of the intensive type and the lack of special equipment for the timely implementation of agrotechnical techniques for sowing, tending, and high-quality harvesting. 

It is advisable to expand the spectrum of legumes and increase the areas under crops that are still insufficiently popular in the Russian Federation: the common bean, chickpea, Indian pea, and lentil, the more so that breeders have created highly productive varieties of these crops suitable for cultivation in various climatic zones of Russia. First of all, this refers to the common vetch, whose seeds are in demand in the Arkhangelsk, Murmansk, Leningrad, and other regions of northwestern Russia and West Siberia (Goncharova, 2020). In recent years, the “State Register…” (2020) has included the common vetch varieties Livenka and Obelna. They are distinguished by high yields of green and dry matter and high protein content at the optimal harvest time: 25–30 %.

In 2018–2020, new broad bean varieties Krasnyi bogatyr’ and Universal and common bean varieties Markiza and Khabarovskaya were obtained. Khabarovskaya was fruit of collaboration with the Far Eastern Research Institute of Agriculture (Khabarovsk). Neither bean variety has foreign analogs, and they are distinguished by plasticity and resistance to abiotic stressors. Their yield potential is 2.5–3.0 t/ha, and they ripen 5–7 days earlier than the standard. The seeds look highly marketable, having appropriate volume, shape, size, and excellent taste. 

The same properties are possessed by lentil grain, which is used in a wide variety of dishes. Unlike peas or beans, lentil seeds are boiled soft 2–3 times faster. Their cooking time is 35–70 minutes. Among the most valuable is the new lentil variety Vostochnaya, bred at FSC LGC. In its development, the biotechnological method of germinating seeds on nutrient media in vitro was used. Vostochnaya is the world’s first lentil variety created with the germplasm of the wild species Lens orientalis.

## Breeding of groats and millet-like crops

Groat crops provide environmentally friendly products suitable for dietary nutrition. Protein content in buckwheat groats varies from 10.9 to 18.9 %, and in the hull, 4 %. Buckwheat groats contain 65–68 % carbohydrates (including 2 % soluble sugars), 12 % proteins, 4 % fat, and 7 % fiber. The compositions of amino acids in the protein and of mineral nutrients determine the dietary value of buckwheat dishes for people of any age. 

The largest areas under groat crops in European Russia are concentrated in the Republic of Bashkortostan and in the Orenburg, Saratov, and Orel oblasts.

Of the group of cereal crops in the world, common buckwheat and millet are most widely used. Less common crops are foxtail, Italian, barnyard, and African millets. Buckwheat and millet, despite their small planting areas, contribute much to the food basket of the population.

In recent years, buckwheat and millet planting areas in the Russian Federation amounted to about 1,700,000 hectares. Both buckwheat and millet are characterized by high variability of gross grain harvest and low dynamics of yield growth. For buckwheat, it varies from year to year from 1.2 to 1.7 t/ha, and for millet, from 1.3 to 1.9 t/ha. 

**Fig. 3.**
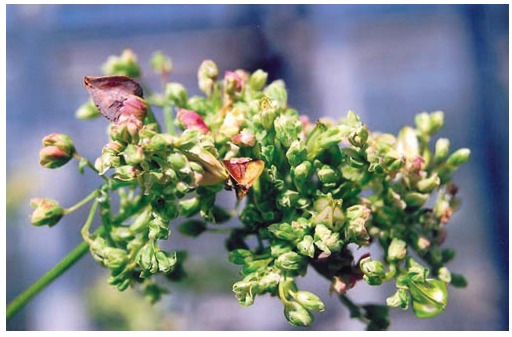
Inflorescence of determinate green-flowered buckwheat with small flowers.

The “State Register…,” contains 52 varieties of buckwheat and 58 varieties of common millet. Only in the last five years, the “State Register…” included five varieties of buckwheat bred at FSC LGC and characterized by the determinate growth type (Fig. 3). Their advantages are even ripening, large (32– 34 g) weight of 1000 grains, high yield of groats (70–74 %), and high protein content (13–16 %) (Zotikov, 2020).

The leading farms of the Orel oblast consistently gather 2.5–3.0 tons of buckwheat per hectare. In recent years, FSC LGC has created a series of high-yielding buckwheat varieties, adapted to a wide range of soil and climatic conditions, capable of producing high grain yields in the field. 

The breeding work on millet is aimed at creating new largegrain, highly productive varieties with a short growing season, resistant to major diseases. Breeding material and varieties of different biotypes differing in ripening terms and differently responding to weather conditions are being created for growing in the main regions of millet cultivation, including northern Russia and Siberia. The Quartet and Sputnik varieties provide high-quality grains when grown in Central Russia and Western Europe (Switzerland and Germany). A grain yield of more than 7 t/ha was recorded for the multilinear variety Quartet and more than 8 t/ha for Sputnik. Alba is the first practically bare-grain variety, not requiring hulling costs (Zotikov, 2020). It has a high content of protein and oil in the grain and is intended for poultry farming. The Kazachye variety combines coarse grain with high yields. It is designed for cultivation in central and southern Russia. The use of biotechnological methods in millet breeding continues. Promising lines of millet dihaploids, new for the Central Federal District forage crops, were obtained: barnyard millet (Echinochloa frumentacea Link.) and African millet (pearl millet, Pennisetum glaucum (L.) R. Br.). The main task of FSC LGC scientists is to develop highly adaptive varieties with high biomass potentials and stable seed yields, resistant to drought and lodging, for use in animal husbandry and poultry farming.

Federal Scientific Center of Legumes and Groat Crops attaches great importance to the creation of varieties that reduce the pesticide load on agroecosystems in crop production, which is a topical issue in the 21st century. This task is mentioned among the main directions in the reports and decisions of the 1992 United Nations Conference: “…increasing plant genetic resistance and good agricultural practices while minimizing the use of pesticides is the best possible option for the future as it guarantees yields, reduces costs, is environmentally friendly and promotes sustainable agriculture...” (Report of the United Nations Conference…, 1992, Chapt. 14).

All new Russian varieties of agricultural crops that are transferred to production carry different genes for resistance to major pests in particular cultivation zones. Moreover, in the modern context, the use of varieties not only resistant to certain races of the main pathogens but also reducing the likelihood of the appearance of their new virulent features, caused by overcoming the crop genetic resistance by the pathogen, becomes a factor of paramount importance. This task is facilitated by using controlled heterogeneity of multilinear varieties and mixtures of varieties (Browning, Frey, 1969). The transition from genetic homogeneity of a phenotypically aligned monoculture to the use of balanced controlled genetic polymorphism for resistance genes reduces the level of existing diseases without chemical treatments of crops (Sidorenko, Vilyunov, 1997; Vilyunov, 2019). 

**Fig. 4.**
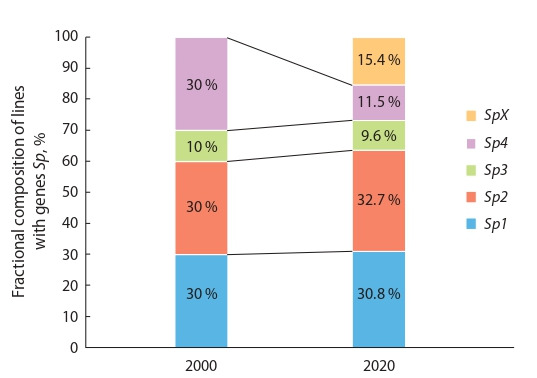
The dynamics of polymorphism for the Sp1–Sp4 genes, which control resistance to smut races, in the multilinear millet variety Quartet for 20 years of growing in the field, when reseeded with its own seeds, without chemical seed treatment. SpX – component requiring additional genetic identification for smut resistance

Studies of the millet variety Quartet, multilinear in resistance to smut, show that for more than 20 years of its cultivation, without chemical treatments against diseases, the variety successfully suppressed the development of smut and completely retained resistance to its local population (Fig. 4), maintaining a high stable yield. Note that for 20 years it retained genetic polymorphism in resistance to all local races of the pathogen (see Fig. 4), close to the initially modeled composition (proportions) of resistant components (lines), which overlapped the racial composition of smut in the Orel region to the greatest extent (Sidorenko, Vilyunov, 1997; Tikhonov et al., 2018; Vilyunov, 2019).

Varieties of many groat crops bred in Russia successfully compete in the world market of environmentally friendly products (Strahm et al., 2019). Russian varieties of millet and buckwheat, when tested in Switzerland, Austria, Germany, gave higher and more stable yields and higher groat quality than local varieties. In particular, the FSC LGC varieties turned out to be more productive and earlier ripening. They were distinguished by better resistance to lodging and diseases. They were characterized by high vigor, which helped them suppress weed growth. They were superior to millet and buckwheat varieties bred in other countries, which did not ripen at all in some years and were harvested only for fodder. The Krupnoskoroe variety has been registered in the EU (Germany), and the registration procedure for the Quartet and Kazachye varieties is underway. The development of the production of organic food (bioproducts) in many countries generated the tendency to arrange process lines for the production of environmentally friendly millet and buckwheat grain and its processing into groats and flour.

To maintain the breeding process at a high level, genetic resources of legumes and groat crops should be mobilized, conserved, and employed. For this purpose, FSC LGC screens more than 20,000 accessions every year. The implementation of the breeding program for the main legumes and groat crops includes

- a comprehensive study of the best varieties and valuable forms and creation of starting material (mutants, regenerants, and recombinants);
- expansion of trait and pre-breeding collections;
- selection of appropriate breeding material, including identification of donors of commercially valuable traits for supporting tasks to solve;
- organization of the breeding process to create high-yielding varieties with resistance to stress and improved product quality, as well as expansion of the genetic base of new original source material at FSC LGC (Zotikov, 2020).

Scientific institutions of the Russian Federation carry out research and solve tasks concerning the Russian and global breeding of legumes and groat crops. Federal Scientific Center of Legumes and Groat Crops carries out a significant amount of research on the development of differentiated resourcesaving systems and technologies for the cultivation of legumes, buckwheat, and millet. It also makes a significant contribution over substantial time and effectively implements governmental programs for breeding and seed production. Federal Scientific Center of Legumes and Groat Crops is the copyright holder and originator of 110 varieties of legumes, groat crops, grain crops, and fodder crops approved for use in various constituent entities of the Russian Federation. It holds 50 patents on breeding achievements in 21 crops, including 28 patents on new adaptive and technological varieties. When raising them, modern achievements of domestic and foreign selection of the Federal Research Center “N.I. Vavilov All-Russian Institute of Plant Genetic Resources” (VIR) weare widely used. Federal Scientific Center of Legumes and Groat Crops maintains its own gene pool collection. Production and environmental tests are carried out using a working collection of genotypes of various agricultural crops having high productivity rates, a complex of commercially valuable traits, and resistance to biotic and abiotic stressors. A significant stock of breeding material is being created. 

In connection with the entry into force of the Federal Law No. 280 of 03.08.2018 “On organic products ...” in 01.01.2020, breeding programs in FSC LGC are included in academic research in order to increase crop productivity, minimize the application of pesticides and mineral fertilizers, and obtain environmentally friendly products (Gryadunova, Khmyzova, 2018).

## Conclusion

The key vectors determining the rise of production of leguminous and groat crops in the Russian Federation involve crop breeding and the development of adaptive technologies. They play an exceptional role in such a rise through the production of certified seeds, which is an economically beneficial way to increase yields and the efficiency of the entire agricultural sector. 

For all that, the main methodological problem of modern breeding is the fullest utilization of recent achievements in fundamental sciences, such as physiology, genetics, biotechnology, biochemistry, immunology, etc. The need for this approach stems from the fact that traditional methods of classical breeding, although used for long, cannot overcome the barrier in the creation of varieties with complex resistance to pests and abiotic stresses. The severest contradiction between productivity, grain quality, and plant resistance to edaphic factors has not been overcome. 

Currently, breeding achievements in studying the mechanisms of plant resistance to drought, low temperatures, and excessive moisture are not used in full. The management of the growing season of new varieties by employing scientifically based crop rotation, as well as using micronutrient fertilizers and growth stimulants, can increase the profitability of crop production. An increase in the areas under legumes and their inclusion in crop rotations will reduce the demand for mineral fertilizers and improve environmental settings in the Russian Federation. In modern conditions of agricultural production, the soybean and pea become key legumes in crop rotations and acquire great national economic importance. The main factor in increasing yields and stabilizing grain production of legumes and groat crops is the creation and accelerated introduction of new early ripening high-yielding varieties of the determinate type, adapted for cultivation in specific climatic conditions of various regions of Russia.

## Conflict of interest

The authors declare no conflict of interest.
